# Discontinuation of follow-up care for young people with complex chronic conditions: conceptual definitions and operational components

**DOI:** 10.1186/s12913-021-07335-x

**Published:** 2021-12-15

**Authors:** Sandra Skogby, Ewa-Lena Bratt, Bengt Johansson, Philip Moons, Eva Goossens

**Affiliations:** 1 Institute of Health and Care Sciences, University of Gothenburg, Gothenburg, Sweden; 2 Department of Paediatric Cardiology, Queen Silvia Children’s Hospital, Gothenburg, Sweden; 3 KU Leuven Department of Public Health and Primary Care, Leuven, Belgium; 4 Department of Surgery and Perioperative Sciences, Umeå University, Umeå, Sweden; 5 Department of Paediatrics and Child Health, University of Cape Town, Cape Town, South Africa; 6 Research Foundation Flanders, Brussels, Belgium; 7 Center for Research and Innovation in Care, Faculty of Nursing, University of Antwerp, Antwerp, Belgium; 8 Department of Patient Care Services, Antwerp University Hospital, Antwerp, Belgium

**Keywords:** Adolescent, Young adult, Chronic disease, Delivery of health care, Continuity of patient care: patient transfer, Lost to follow-up

## Abstract

**Background:**

A substantial proportion of young people with Complex Chronic Conditions (CCCs) experience some degree of discontinuation of follow-up care, which is an umbrella term to describe a broken chain of follow-up. Discontinuation of follow-up care is not clearly defined, and the great plethora of terms used within this field cannot go unnoticed. Terms such as *“lost to follow-up”, “lapses in care”* and *“care gaps”*, are frequently used in published literature, but differences between terms are unclear. Lack of uniformity greatly affects comparability of study findings. The aims of the present study were to (i) provide a systematic overview of terms and definitions used in literature describing discontinuation of follow-up care in young people with CCC’s; (ii) to clarify operational components of discontinuation of follow-up care (iii); to develop conceptual definitions and suggested terms to be used; and (iv) to perform an expert-based evaluation of terms and conceptual definitions.

**Methods:**

A systematic literature search performed in PubMed was used to provide an overview of current terms used in literature. Using a modified summative content analysis, operational components were analysed, and conceptual definitions were developed. These conceptual definitions were assessed by an expert panel using a survey.

**Results:**

In total, 47 terms and definitions were retrieved, and a core set of operational components was identified. Three main types of discontinuation of follow-up care emerged from the analysis and expert evaluation, conceptually defined as follows: ***Lost to follow-up care*:** “*No visit within a defined time period and within a defined context, and the patient is currently no longer engaged in follow-up care”;* ***Gap in follow-up care***: “*Exceeded time interval between clinic visits within a defined context, and the patient is currently engaged in follow-up care”; and* ***Untraceability***: “*Failure to make contact due to lack of contact information”.*

**Conclusion:**

By creating a common vocabulary for discontinuation of follow-up care, the quality of future studies could improve. The conceptual definitions and operational components provide guidance to both researchers and healthcare professionals focusing on discontinuation of follow-up care for young people with CCCs.

**Supplementary Information:**

The online version contains supplementary material available at 10.1186/s12913-021-07335-x.

## Introduction

Complex chronic conditions (CCCs) of childhood onset comprise a wide spectrum of conditions, ranging from mild to severe complexity. Within paediatric and adolescent health, CCCs are defined as “*any medical condition that can be reasonably expected to last at least 12 months (unless death intervenes) and to involve either different organ systems or one organ system severely enough to require specialist paediatric care and probably some period of hospitalization in a tertiary care centre*” [1]. Patients with CCCs cannot be considered cured, considering substantial risks of long-term complications. Hence, life-long medical follow-up care is required, including transfer of care from paediatric to adult focused healthcare facilities.

Despite the required lifelong medical follow-up, a substantial proportion of patients experience some degree of discontinuation of follow-up care [2–22] . Discontinuation of follow-up care is an umbrella term, covering many different terms used in literature describing a disrupted chain of follow-up care. The transfer from paediatric to adult healthcare facilities is deemed a particularly vulnerable phase. For example, a review showed that 11–24% of young people with diabetes type 1 did not continue follow-up care [18]. One study in young adults with Turner syndrome reported 12.7% not being under regular follow-up [21] and one study in young people with congenital adrenal hyperplasia reported 50% being lost to follow-up [19]. For young people with juvenile idiopathic arthritis, one study reported 52% unsuccessful transfer [20]. Many reports concern young patients with congenital heart disease, using a plethora of terms for the reported proportions ranging from 3.6–62.7% [22]. Discontinuation of follow-up care is associated with adverse outcomes such as increased morbidity, hospitalizations and urgent interventions and re-interventions [9, 18, 23, 24] and therefore requires active prevention.

However, discontinuation of follow-up care is not a universal term nor clearly defined. The great plethora of terms used in this field cannot go unnoticed. Terms such as *“lost to follow-up”, “lapses in care”, “care gaps”*, and *“lack of follow-up”* are frequently used in published literature, but the conceptual difference between these terms is not currently defined. Lacking uniformity regarding these terms and definitions greatly affects study comparability since it significantly contributes to methodological differences. The need for uniformity of terms and definitions is important, both from a research and clinical perspective, with patients, healthcare professionals and scientists benefiting from a clarification of terminology.

The aims of the present study are to:

(i) Provide a systematic overview of terms and definitions used in literature for describing discontinuation of follow-up care for young people with complex chronic conditions.
(ii) Clarify operational components of discontinuation of follow-up care.
(iii) Develop conceptual definitions and propose terms for discontinuation of follow-up care.
(iv) Perform an expert-based evaluation of the developed terms and conceptual definitions.

## Methodology

### Study design

As a first step, a systematic literature search was performed to provide an overview of terms and definitions related to the concept of discontinuation of follow-up care that are used in literature. As a second step, a modified version of summative content analysis [25] was used for analysis of included definitions and development of initial conceptual definitions. In the third step, an expert panel provided input on initial conceptual definitions through a survey, which guided the final formulation of conceptual definitions.

### Procedure

#### Literature search

**Fig. 1:**
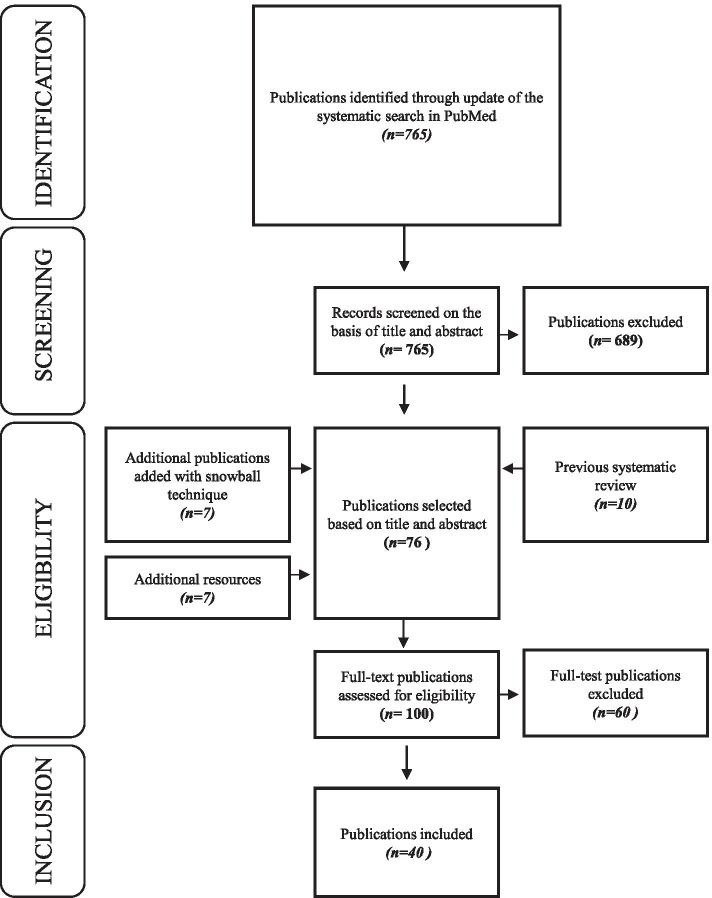
Prisma flowchart of the study selection process

Starting with 10 publications identified in a previous systematic review investigating predictors of care gaps [26], an updated systematic search was performed in PubMed, using the search string of the same systematic review [26]. The search was limited to the period October 1, 2014 until October 29, 2018 to capture articles published after the systematic review [26]. Publications were selected that met the following criteria: (i) primary research, (ii) study population including young people (aged 10-25y) diagnosed with CCCs, (iii) study aim (partly) focused on discontinuation of follow-up care, and (iv) published in English. Editorials, published comments, and letters to the editor were excluded. Screening of eligible publications was performed by the first and last author independently. In total, 40 publications were included for analysis [2–9, 14, 17, 19, 20, 27–54] including 10 publications from the previously published systematic review [26], 16 publications from the updated systematic search described above, 7 publications using snowball-sampling techniques and 7 publications from additional resources (publications known by the research group which did not appear in the systematic search). (Fig. 1).

#### Data analysis

Data analysis was performed using a modified summative content analysis approach, inspired by Hsieh and Shannon’s methodological description [25]. This type of analysis is suitable for exploring how words are used within texts, and to gain an understanding of the contextual use of specific words or content. It includes quantification of terms and content within the text, as well as interpretation of the underlying meaning [25]. This analysis technique focused on the manifest content. However, when performing this type of content analysis, interpretations are made by the researcher [55]. To clarify operational components of discontinuation of follow-up care, an inductive approach was used. Operational components can be described as those parts of an operational definition which provides instruction for how a concept should be measured. It could, for example be a section or combination of words in a definition implying measurement trough time, or through a specific event or a section implying the importance of a specific health care context or medical evaluation when investigating a concept.

**Fig. 2:**
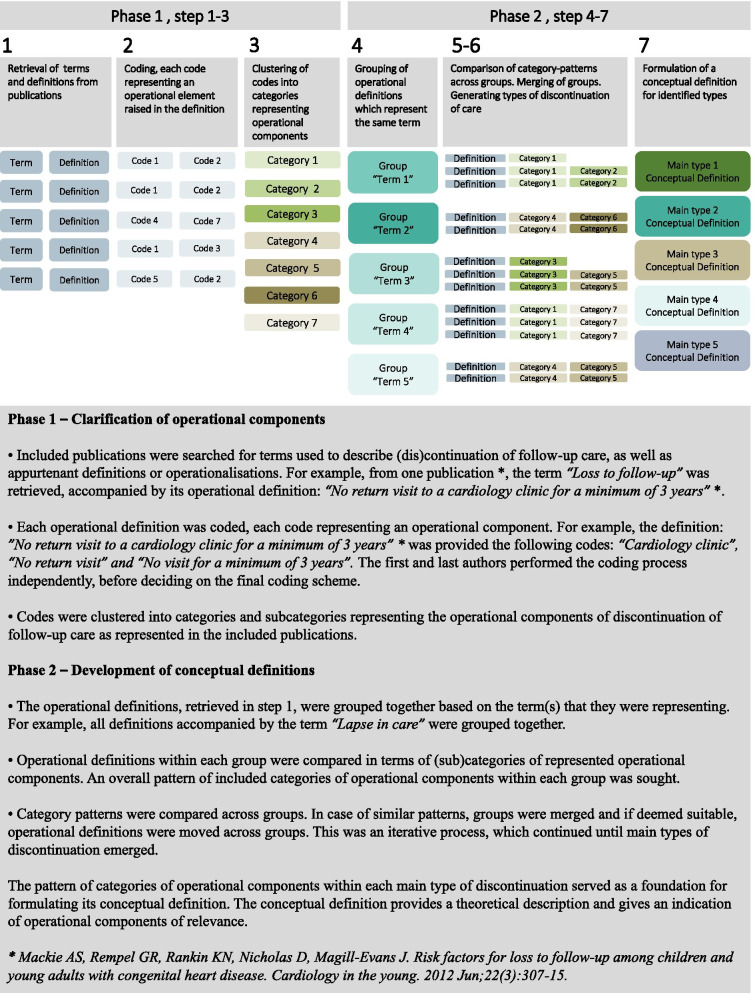
Flowchart of the analysis process

The analysis process was carried out in seven steps, divided into two phases (see Fig. 2). In phase 1, terms and operational definitions were retrieved from the selected literature. Definitions were given several codes based on the included operational components. These codes were subsequently clustered into categories of operational components based on overlapping content. (Step 1–3). In phase 2, the operational definitions were grouped based on the terms they represented. Definitions within and across groups were compared in terms of operational components. This iterative process resulted in preliminary types of discontinuation. (Step 4–6). A type of discontinuation can be described as a recurring combination of operational components. A preliminary conceptual definition for each respective type was formulated based on included operational components. (Step 7). Each preliminary type was appointed a preliminary term. A term is a word or a sentence, functioning as a label.

The data extraction of terms and definitions was performed by two independent researchers (the first and the last author) and included repeated reflections and discussions. The subsequent analysis was performed jointly by the first and last author.

#### Evaluation of conceptual definitions and selection of terms

Experts, including authors of included publications and researchers active within the field, were approached to evaluate the proposed conceptual definitions and to suggest suitable terms for each respective conceptual definition accordingly. This expert evaluation was performed through completion of a survey (Supplementary material-survey) whereby participants were asked to link each conceptual definition to a suitable term. A list of terms was provided in the survey, as well as free space to suggest alternative terms or to provide additional comments. Terms suggested by the authors of the present study were not included in the survey. After excluding the authors of the present study, all authors of the included publications were invited to participate through their email address for correspondence mentioned in the publication. Additional active researcher within the field known by the research group (*n* = 3) were also invited to participate. In total, 40 experts were invited to participate and 12 (30%) agreed to participate. Agreement percentage for each definition were calculated, and along with free space comments, constituted a basis for discussion and further development of the conceptual definitions within the research group. An agreement percentage of > 70% was considered indicative of agreement.

## Results

### Terms and definitions

In total, 47 terms with accompanying operational definitions were retrieved from the included publications (Table 1). Of these, 26 terms were considered to be unique (55%). Only six terms occurred more than once. These were “*Lost to follow-up”*, “*Loss of/to follow-up”*, “*Successful transfer”*, “*Successful transition”*, “*Lapse in/of care”* and “*Retention in care*”.

**Table 1:** Overview of terms and definitions

**Gleeson et al., 2013** [19]	Lost to follow up	stopped attending either paediatric or adult clinics or were discharged because of nonattendance before care could be transferred to another adult service
**de Bono et al., 2005** [30]	Lost to follow up	not being seen in any cardiac clinic for a period of at least 2 years
**Wacker et al., 2005** [53]	Lost to follow-up	failed to return for a scheduled FU visit for > 5 years
**Wray et al., 2013** [54]	Lost to specialist follow up	not been seen within any specialist network in the past 3 years
**Gerardin et al., 2016** [32]	Lost to cardiology follow-up	Not seen a cardiologist in an outpatient clinic during the 3 year study period
**Bohun et al., 2016** [2]	Lost to follow up	Not seen by any provider in the institution
**Agwu et al., 2015** [27]	Lost to follow-up	No primary HIV outpatient provider visits during the 1 year (365 days) after the 22nd birthday.
**Trefz et al., 2015** [48]	lost to follow-up	≥3 times no show for outpatient evaluation and/or no blood samples sent for Phe analysis” (Phe = phenylalanine)
**Kakkar et al., 2016** [36]	Lost to follow-up	could not be reached
**Sawicki et al., 2017** [44]	lost to follow-up	patient without any data in the Registry
**Mackie et al., 2009** [6]	Loss of follow-up	Patients who were not seen by a cardiologist within the indicated age range but were seen again by a cardiologist in an older age group or patients who had their last cardiology follow-up at that age.
**Mackie et al., 2012** [40]	Loss to follow-up	no return visit to a cardiology clinic for a minimum of 3 years
**Pyatak et al., 2017** [41]	Loss to follow-up	The number of routine diabetes care visits (including both paediatric and adult care visits) at the study’s participating clinics during the 12-month study period.
**Sam-Agudu et al., 2017** [42]	Retention in care	Having made at least two clinic visits separated by a 6-month period within 12 months and at least four visits each separated by at least 6 months within 24 months post transfer
**Steinbeck et al., 2015** [45]	Retention in adult services	The definition of retention in the adult service was: (i) the participant continued to be a patient of the adult diabetologist they were originally referred to; or failing that (ii) the participant successfully transferred to another adult diabetologist
**Norris et al., 2013** [7]	Retention in care	any cardiology clinic visit within 2 years of the telephone interview
**Gurvitz et al., 2013** [5]	Gap in cardiology care	more than 3 year interval between any cardiology appointments (internal medicine, paediatric or adult congenital cardiology)
**Sawicki et al., 2017** [44]	Gap in care	time in days between last recorded encounter at a paediatric or affiliate program and first recorded encounter at an adult program
**Sawicki et al., 2017** [44]	Prolonged gap in care	A gap in care in accredited CF centres of greater than or equal to 365 days
**Garvey et al., 2012** [31]	(time) Gap	describing post transition gaps in care > 6 months for patients with type 1 diabetes
**Mackie et al., 2016** [39]	Excess time between paediatric and ACHD care	The time interval (in months) between the final paediatric visit and the first ACHD visit, minus the recommended time interval between these visits
**Wisk et al., 2015** [51]	Transfer gap	time from the last paediatric-focused PCP visit to the first adult-focused PCP visit
**Norris et al., 2013** [7]	Lapse in care	Any 2y interval without cardiac care
**Valente et al., 2013** [49]	Lapse of care	no direct recorded contact with our adult congenital heart disease (ACHD) centre within the last 3 years
**Yeung et al., 2008** [9]	Lapse in medical care	Length of time from leaving care at a paediatric institution to receiving subsequent cardiac care at any institution. A duration since last visit greater than the 2-year
**Sattoe et al., 2017** [43]	Successful transition	Indicator 1 – patient not lost to follow-up: It is recorded whether a patient is transferred and to where, and/or a note or letter of transfer of the patient to adult care is found in the electronic patient record (EPR) (yes/no). Those who score ‘no’ are no longer seen in paediatric care, but it is not clear whether and where they receive adult care treatment. • Indicator 2 – attending scheduled visits in adult care: The patient has not missed any consultations in the 3 years after transfer (yes/no), as reported in the EPR. • Indicator 3 – patient building a trusting relationship with adult provider: The patient trusts the current adult care provider as indicated by a score > 15 on a scale of 5–20 (yes/ no) in the survey. A five-item 4-point Likert scale (from 1 = “never” to 4 = “always”; α = 0.90) was used. This was measured in the questionnaire with a validated Dutch adaptation of one scale from the American Consumer Assessment of Health Plan Surveys questionnaire (Delnoij et al. 2006)
**Andemariam et al., 2014** [28]	Successful transition	attendance of at least one outpatient visit at the adult SCD centre after being discharged from the paediatric SCD program
**Bohun et al., 2016** [2]	Successful transfer	attending at least one adult congenital heart disease clinic visit
**Vaikunth et al., 2018** [17]	Successful transfer	Transfer of care was defined as successful if patients seen in the transition clinic were subsequently seen on at least one occasion in the ACHD clinic at the adult hospital
**Harbison et al., 2016** [14]	Successful transfer	The subsequent attendance at adult cardiology within 2 years of PC visit
**Hazel et al., 2010** [20]	Unsuccessful transfer	failure to make initial contact with an adult rheumatologist, or failure to continue to follow-up with an adult rheumatologist 2 years after transfer (no contact for a 1 year period after the last scheduled appointment)
**Wisk et al., 2015** [51]	Transfer timing	time to first visit with an adult focused PCP
**Reid et al., 2004** [8]	Successful transfer	Attended at least 1 appointment of any type (e.g., clinic, echocardiogram, cardiac catheterization, or surgical) at a CACH centre. (CACH = Canadian Adult Congenital Heart)
**Goossens et al., 2011** [4]	No follow-up	currently not in cardiac follow-up or if they could not be contacted by mail or phone
**Wojciechowski et al., 2002** [52]	Uninterrupted care	whether or not the participant kept his or her initial ACC appointment and the length of time between the last PCC appointment and the first ACC appointment
**Goossens et al., 2015** [3]	Not being in cardiac follow-up	A complete cessation of cardiac care was confirmed
**Arthur et al., 2018** [29]	Continuity of primary care	Concentration of visits with a single provider or team in primary care
**Hattori et al., 2016** [34]	Ended or interrupted follow-up	No transfer from paediatric care or ended or interrupted follow-up by paediatric renal services, but later presented to adult renal services without medical, social, and/or educational information prepared by paediatric renal services
**Kakkar et al., 2016** [36]	Engaged in care	at least one physician visit within 6 months of the interview
**Kayle et al., 2018** [37]	Continuity of care	the frequency of clinic appointments and mean duration in care in years
**Stringer et al., 2015** [46]	Patient compliance with follow-up	Ongoing care with adult rheumatologic follow-up after transfer of care
**Suris et al., 2015** [47]	Attending scheduled visits in adult care	Attending scheduled visits in adult care: no missed consultations unless previously cancelled and rescheduled.
**Hankins et al., 2012** [33]	Fulfilment of first appointments	went for their first appointment with the adult SCD provider within 3 months of leaving paediatric care
**Holmes-Walker et al., 2007** [35]	Attendance at specialist clinic	The aim was to ensure a minimum of two visits per year to the service
**Kipps et al 2002** [38]	Regular clinic attendance	Regular clinic attendance rates (at least 6 monthly) from 2 years pretransfer to 2 years post-transfer
**Steinbeck et al., 2015** [45]	Engagement in adult services	(i) at least one visit to an adult diabetes service post-discharge from paediatric care; (ii) frequency of visits to the adult service; and (iii) the time interval between the last paediatric diabetes service visit and first adult diabetes service visit
**Van Walleghem et al., 2008** [50]	Drop out	first year fall-out rate after transfer from paediatric to adult care

### Operational components

**Fig. 3:**
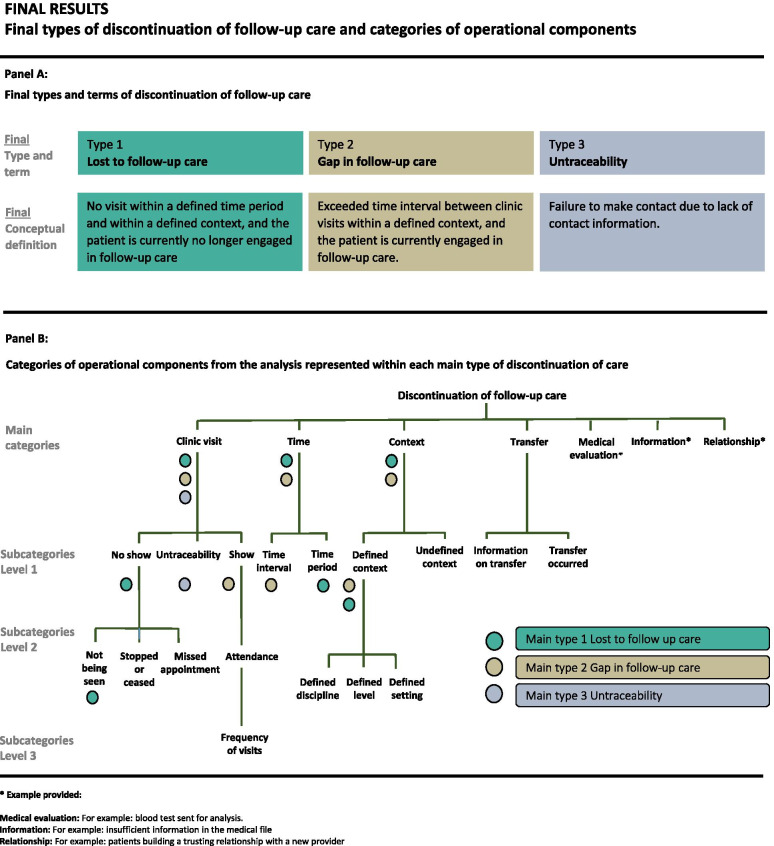
Final types of discontinuation of follow-up care and categories of operational components

In total, seven main categories of operational components were identified. These respective components were labelled *Clinic visit*, *Time*, *Context*, *Transfer*, *Medical evaluation*, *Information* and *Relationship*. (Fig. 3, panel B).

The category ‘*Clinic visit’* was subdivided into three subcategories, including ‘*No show’*, ‘*Show’* and ‘*Untraceability’.* In addition, the subcategories, ‘*No show’* and ‘*Show’* were further divided, (see Fig. 3, panel B*).*

The category ‘*Time’* was divided into two subcategories, ‘*Time interval’* and ‘*Time period’.*

The category ‘*Context’* was subdivided into the subcategories ‘*Defined context’* and ‘*Undefined context’.* The subcategory, ‘*Defined context’*, was further divided, (see Fig. 3, panel B*).*

The category ‘*Transfer’* was subdivided into the subcategories ‘*Information on transfer’* and ‘*Transfer occurred’.* (see Fig. 3, panel B).

### Conceptual definitions and expert evaluation

Initially, five distinct types of discontinuation emerged from the analysis. The preliminary terms for types 1–5 were (1) *Lost to follow-up*, (2) *Retention in care*, (3) *Gap in follow-up care*, (4) *Unsuccessful transfer* and (5) *Untraceability* (Table 2). The five preliminary types were presented to the expert panel. Agreement was reached for three types: type 2 *Retention in care*, type 4 *Unsuccessful transfer* and type 5 *Untraceability* (Table 2). Five experts provided free text comments. These respective comments included reflections on “transition” being predominantly a contextual factor, and the importance of considering whether or not the patient has re-engaged in care.

**Table 2:** Preliminary conceptual definitions presented to the experts-panel

**Preliminary type**	Type 1	Type 2	Type 3	Type 4	Type 5
**Preliminary term**	Lost to follow-up	Retention in care	Gap in follow-up care	Unsuccessful transfer	Untraceability
**Preliminary conceptual definition**	No show or not being seen for a clinic visit within a defined time period and within a defined context	Attending a clinic visit within a defined time period and within a defined context	A defined time interval between clinic visits within a defined context	Not attending a clinic visit within a defined context after transfer	Failure to make contact due to lack of information
**Agreement percentage**	50%	91%	42%	75%	83%

After considering agreement rates, suggested terms and free text comments from experts, type 2 *Retention in care* was omitted, due to perceived conceptual overlap with type 1 *Lost to follow-up care* and for being considered more related to continuity of follow-up care rather than discontinuation of follow-up care. In addition, type 4 *Unsuccessful transfer* was omitted due to transfer being considered a contextual factor rather than a distinct type.

The three remaining types, type 1 *Lost to follow-up*, type 3 *Gap in follow-up care* and type 5 *Untraceability* were reformulated based on comments from the experts and discussions within the research group, resulting in three final types of discontinuation of follow-up care (Fig. 3, panel A).

## Discussion

### Broad range of terms and definitions

This study identified a broad range of terms and definitions being used in the literature in relation to (dis)continuity of follow-up care in young people with CCCs. Indeed, a published systematic review on predictors of care gaps [26] described a range of definitions used in literature, as well as broad variation in the level of detail of these definitions [26]. The findings of the present study demonstrated that as few as six terms reoccurred and despite a broad range of definitions and terms, a core set of components could be identified among the definitions. Similarities across terms and definitions were found to be greater than the differences.

### Conceptual and operational definitions

The difference between an operational and conceptual definition needs to be considered when interpreting the results of this study. A conceptual definition comprises a formulation of abstract and/or theoretical meaning [56], whereas an operational definition entails a formulation of the procedures for measurement [56]. The definitions of the three types of discontinuation of follow-up care, as identified in the present study, should be considered as conceptual definitions and can be broadly applied to young people with CCC’s, irrespective of their condition. However, conceptual definitions need to be operationalized in order to scientifically measure and describe the concept. To convert the identified conceptual definitions towards operational definitions, a more detailed operationalization is required, enabling the measurement of this type of discontinuation in a defined patient population, both in clinical practice and for research purposes. For example, periods and intervals of time can be operationalized by specifying them in terms of a context, setting and study population, as these aspects often vary according to the condition or disease complexity.

For example, *Lost to follow-up care* was conceptually defined as: “*No visit within a defined time period and within a defined context and the patient is currently no longer engaged in follow-up care.”* Based on guidelines for complex congenital heart disease (CHD) [57–61], this type of discontinuation could be operationalized as follows: “*No visit to a specialist adult CHD clinic within a 12-month period and the patient is currently no longer engaged in follow-up care.”* However, when investigating lost to follow-up in a different patient population, such as adolescents with diabetes mellitus, type 1, the operational definition, as defined by recent guidelines [62], could instead be: *“No visit to a paediatric diabetes-clinic within a 3-month period and the patient is currently no longer engaged in follow-up care* “.

When designing a research study, additional operational components to those included in the conceptual definition could be of relevance if they aim to integrate a specific context, setting or disease phase. For example, the element of *Transfer* was included in many of the definitions derived from the literature but was not included in any of the conceptual definitions of the three main types. After considering comments from experts, transfer was deemed a contextual factor not indicating a distinct type of discontinuity and was therefore omitted. However, for a study conducted during the period of transfer of care, a transfer component should be added as an operational element of the definition. For example, *Gap in follow-up care* was conceptually defined as:*” Exceeded time interval between clinic visits within a defined context, and the patient is currently engaged in follow-up care.”* An investigation of *Gaps in follow-up care* during a care transfer could, for example, be operationalized as: *“A time interval exceeding 3 years between paediatric and adult clinic visits, and the patient is currently engaged in follow-up care.”*

Being “engaged in follow-up care” or not is an operational component included in both type 1 *Lost to follow-up care* and type 2 *Gap in follow-up care*. In this context, being “engaged in follow-up care” at large refers to a current connection or interaction between the patient and the health care system. Operationally, it could for example refer to attendance of clinic visits. A full operationalization of type 1 *Lost to follow-up care* could then for example be: “*No visit to a specialist adult CHD clinic within a 12-month period and the patient is currently not attending outpatient clinic appointment.*

### Operational components

Seven main categories of operational components emerged from the analysis. To provide an example, the term *“Loss to follow-up”* was retrieved from one of the included publications [40], accompanied by its operational definition: *“No return visit to a cardiology clinic for a minimum of 3 years”* [40]. This definition was given the following codes: *“Cardiology clinic”, “No return visit” and “No visit for a minimum of 3 years”.*

Codes were then clustered into categories and sub-categories of operational components. For example, the code *“No visit for a minimum of 3 years”* was placed in the main category “*Time*” which was subdivided into two subcategories, “*Time interval”* and “*Time period”*.

The subcategory “*Time period”* consisted of codes such as *“No visit for a minimum of 3 years”* or “*not seen for a period of at least 2 years*”. In contrast, the subcategory “*Time interval”* consisted of codes such as “*time between the final paediatric visit and the first adult visit*” and “*more than 3 years interval between appointments*”.

The operational components represent essential parts of an operational definition. When an operational component is specified it provides instructions for measurement and thereby plays an important role when translating a conceptual definition into an operational definition. For example, type 2 ‘*Gap in follow-up care’* was conceptually defined as: “*An exceeded time interval between clinic visits within a defined context, and the patient is currently engaged in follow-up care”.* This conceptual definition comprises the following operational components: *Time interval*, *Defined context*, *Show (for a clinic visit) and Being engaged in follow-up care* (Fig. 3, panel B). These respective operational components do provide guidance towards formulation of an operational definition.

### Applying conceptual definitions and operational components

All terms and concepts used in the literature should be conceptually defined to enhance clarity and comparability of study results. We suggest the application of a stepwise approach enhancing conceptual clarity for research purposes when aiming to investigate discontinuation of follow-up care in young people with CCCs.

1. Conceptually define the concept by providing a thorough theoretical explanation. *(The three types of discontinuation of follow-up care, with conceptual definitions and suggested terms (*Fig. 3*, panel A), can be of guidance.)*
2. Choose an appropriate term for the concept. Consider alternative uses and semantics of the term in relation to your conceptual definition. *(If using one of the conceptual definitions from the present study or a modified version, please use the suggested term in order to increase comparability in a longer perspective.)*
3. Determine operational components of relevance for the study. Consider contextual factors as well as aspects of timing and procedures of measurement. Make sure that relevant operational components are included in your conceptual definition. *(The categories of operational components (*Fig. 3*, panel B) can provide guidance, but are not to be considered comprehensive.)*
4. Operationally define the concept by specifying the operational components in the conceptual definition. To operationalize the components, replace the theoretical explanation with clear measurement instructions. Make sure that these operationalization and measurements are in line with condition- and context-specific recommendations or guidelines.
5. Provide both a conceptual and operational definition of the concept in the method section. Provide the operational and conceptual definition separately.

### Related concepts

When considering discontinuation of follow-up care from a broader perspective, a related concept could indeed be Continuity of Care (CC). In conformity with discontinuation of follow-up care, CC is a concept which is “often presumed rather than stated” [63]. One definition [64] presents three main types, and two core elements. The three types of CC are: “*Informational continuity”*, referring to information use and personal circumstances in order to provide appropriate care. “*Relational continuity”,* referring to the therapeutic relationship, and “*Management continuity”*, referring to consistent and coherent approaches to management of care, which is also responsive to the changing needs of a patient [64]. Relational and information aspects can be found among the identified operational components in the present study, but these aspects are not included in any of the main types of discontinuation. This reflects how these aspects are seldom investigated within this field as compared to more management-related aspects, such as time intervals and the context for follow-up care. One could argue that in relation to the different types of CC, the identified types of discontinuation of follow-up care in the present study, are mostly related to “*Management continuity”*. However, the definition of “*Management continuity”*, puts emphasis on the care management across different providers [64] which is not the case for the definitions in the present study, unless the transfer component is included. The two core elements of CC are “*received and experienced by an individual*” and” *care provided over time”* [64]. In addition, CC can also be viewed from either a *person-focused* or a *disease-focused perspective* [63]. The aspect of *time* is central in two of the main types identified in the present study. However, in contrast to CC, the different types of discontinuation do not consider the individual patient experience, and the definitions could be considered as mainly diseased-focused.

It is important to acknowledge that the findings of the present study are a reflection of the current body of published work within this field. The authors do not suggest setting aside other aspects, such as patient experiences, person-focused perspectives or aspects of information and relations, which are emphasized within the definition of CC. Based on the present findings, it could rather be suggested that more studies illuminating the young peoples’ perspectives are needed within our research field and that the identified types of discontinuation of care should be considered as types rather than a comprehensive typology.

## Methodological considerations

There are many possible choices in terms of analysis method for this type of study. We choose a modified summative content analysis approach. Conceptual analysis methods could also have been an option. However, conceptual analysis often requires broader literature searches to cover all possible uses of a concept, sometimes from different disciplines or contexts [65]. In addition, useful conceptual analysis requires a very rigorous process and ideally an intention of expanding theory within a discipline [65]. The present study is limited to a specific context and does not attempt to cover all possible uses of terms and concepts.

Please consider that the focus of this study was discontinuation of follow-up care for young people with CCCs. If the literature search had included other patient populations, the identified operational components might have differed.

Furthermore, no conceptualizations in this study should be considered final, as they are all shaped by the present, and later on, as a natural consequence of new knowledge and emerging perspectives they might be discredited or in need of revision [56].

The strengths of the study are the systematic inclusion of publications, as well as the evaluation of conceptual definitions and suggested terms by experts within the field. However, some methodological limitations ought to be considered. Firstly, the response rate of the survey was quite low and additional input from experts could have further strengthened the level of consensus. Secondly, the literature search was performed using only one database, which could be considered a limitation. Thirdly, the search string did not cover all types of terms usage, such as *“retention in care”* or *“lapses in care”*, however, the search string did include overarching terms such as “continuity”, “continuum”, “transfer” and “transition” which are often used in combination with other terms when describing discontinuation. In addition, the literature search was extended using snowball techniques and additional resources.

## Future research

Future research would ideally investigate the conceptual meaning of discontinuation of follow-up care across different patient populations, which might complement the current findings and contribute to further conceptualization and clarity of the phenomenon of discontinuation. Attempts to improve consensus on condition-specific operationalizations would also be of value, enhancing comparability of future study findings. Additionally, more studies illuminating the young peoples’ perspectives on discontinuation of follow-up care is clearly needed.

## Conclusion

Providing conceptual definitions in combination with operationalizations that are in line with condition- and context-specific guidelines or recommendations can enhance comparability of study findings in the future. Despite a broad range of definitions and terms found in the literature, a core set of operational components and three main types of discontinuation of follow-up care could be identified. The three main types with their conceptual definitions and the identified operational components can provide guidance when designing research investigating discontinuation of follow-up care for young people with CCCs. Attaining complete uniformity within this field is probably optimistic. However, increased awareness of the use of specific terms and definitions is an important step forward in attaining conceptual clarity. By creating a common vocabulary for discontinuation of follow-up care, the quality of future studies could improve, and dissemination of research findings be eased.

## Supplementary Information

**Additional file 1.**

## Data Availability

The data used and analysed during the current study are available from the corresponding author on reasonable request.

## Abbreviations

CCCs: Complex chronic conditions
CHD: Congenital heart disease
CC: Continuity of care
