# Supplementary material for: Discontinuation of follow-up care for young people with complex chronic conditions: conceptual definitions and operational components
Source: BMC Health Serv Res. 2021 Dec 15;21:1343. doi: 10.1186/s12913-021-07335-x (PMC8672472; doi:10.1186/s12913-021-07335-x)
Supplement: Supplementary file 1 — Additional file 1. [file 12913_2021_7335_MOESM1_ESM.pdf]

I consent to answer the questions below ☐

Please, link the five different conceptual definitions to suitable terms. Select a term for each definition, using the drop-down lists.

Please, feel free to write down any ideas, thoughts or comments to your answers.

Terms		Conceptual definitions for five types of discontinuation of care
Untraceability	Select<br>Comments:	No show or not being seen for a clinic visit within a defined time period and within a defined context
Gap in care	Select<br>Comments:	Attending a clinic visit within a defined time period and within a defined context
Unsuccessful transfer	Select<br>Comments:	A defined time interval between clinic visits within a defined context
Lost to follow-up	Select<br>Comments:	Not attending a clinic visit within a defined context after transfer
Retention in care	Select<br>Comments:	Failure to make contact due to lack of information

If you have further comments, please write them here:
